# Supplementary material for: Accurate Label-Free Quantification by directLFQ to Compare Unlimited Numbers of Proteomes
Source: Mol Cell Proteomics. 2023 May 22;22(7):100581. doi: 10.1016/j.mcpro.2023.100581 (PMC10315922; doi:10.1016/j.mcpro.2023.100581)
Supplement: Supplemental Figures S1–S4 [file mmc1.pdf]

SUPPLEMENTARY FIGURES

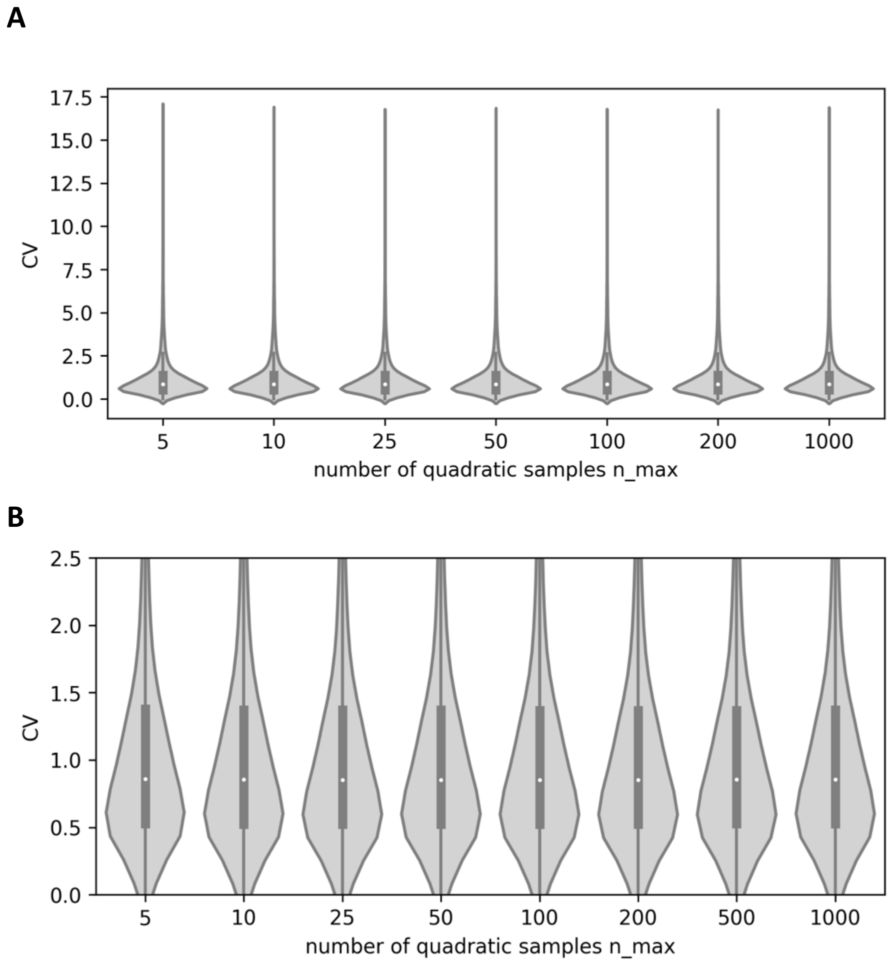

Supplementary Figure 1: A) CVs over precursors of the yeast interactome datasets with different numbers of samples n_{max} used for creating an average trace as described in the main text. No visible change depending on n_{max} can be seen in these distributions, indicating that a high n_{max} is not necessary for improved performance. B) Zoom in.

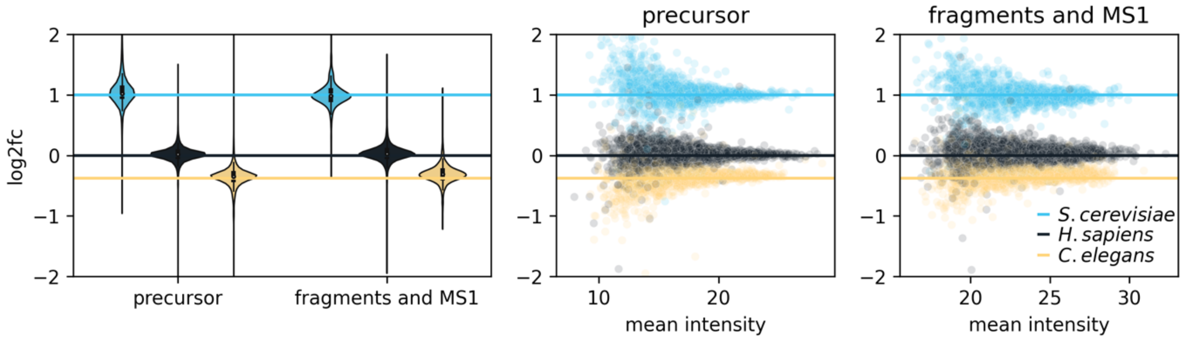

Supplementary Figure 2: Comparing protein quantification in DIA based on precursor as well as fragment and MS1 level data. The underlying dataset is the mixed species experiment of **Figure 3C** and described in the main text. In the case of precursor-level data, the directLFQ intensity traces are derived from the "FG.Quantity" column in the Spectronaut report, which gives one intensity per charge state and potentially modified peptide sequence. The fragment and MS1 level data assigns one intensity trace to each individual fragment ion ("F.PeakArea") and to each MS1 isotope intensity ("FG.MS1IsotopeIntensities (Measured)"). Overall, both results show good quantification, with proteins meeting the expected fold changes, but the fragment seems to be more consistent. For this reason, we chose the fragment approach for DIA data analysis in this study. The user does however have the option to analyze based on precursor information.

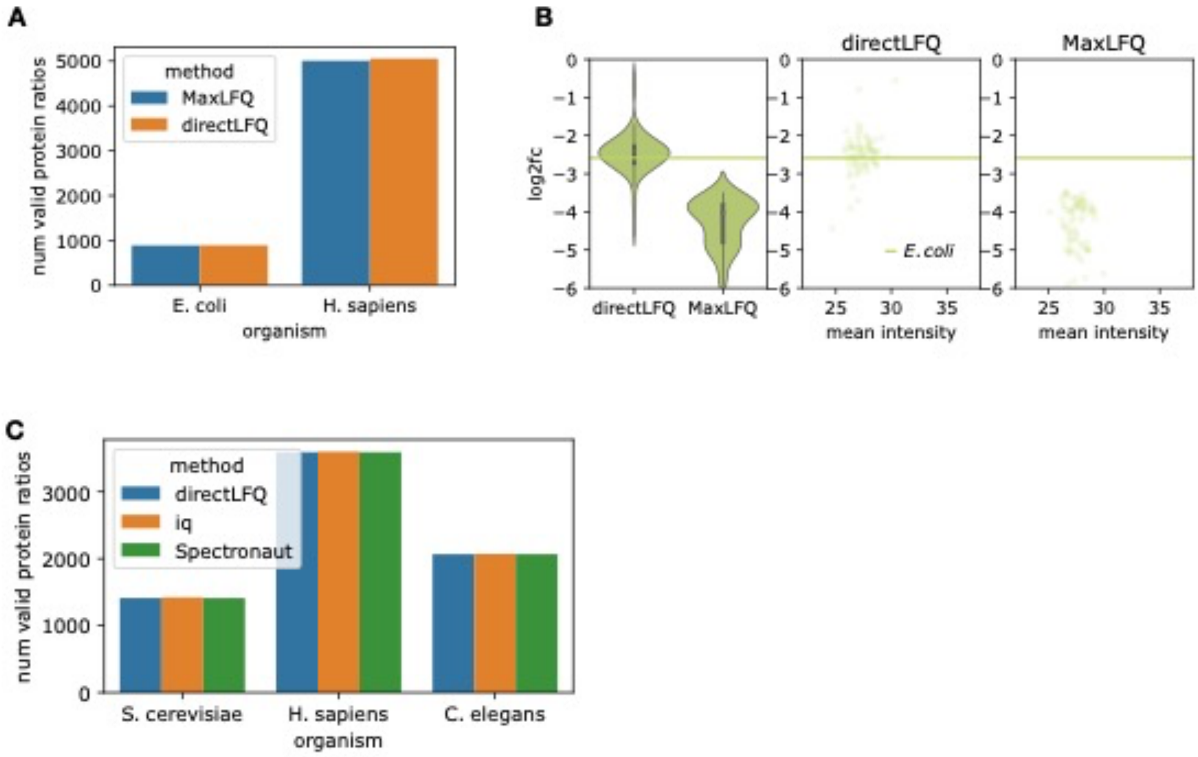

Supplementary Figure 3: Details on the ratio plots. A) Number of proteins ratios in the DDA ratio comparison displayed in **Figure 3A**. B) Quantification of outlier proteins in MaxLFQ und comparison to directLFQ. C) Number of protein ratios in the DIA ratio comparison displayed in **Figure 3B**.

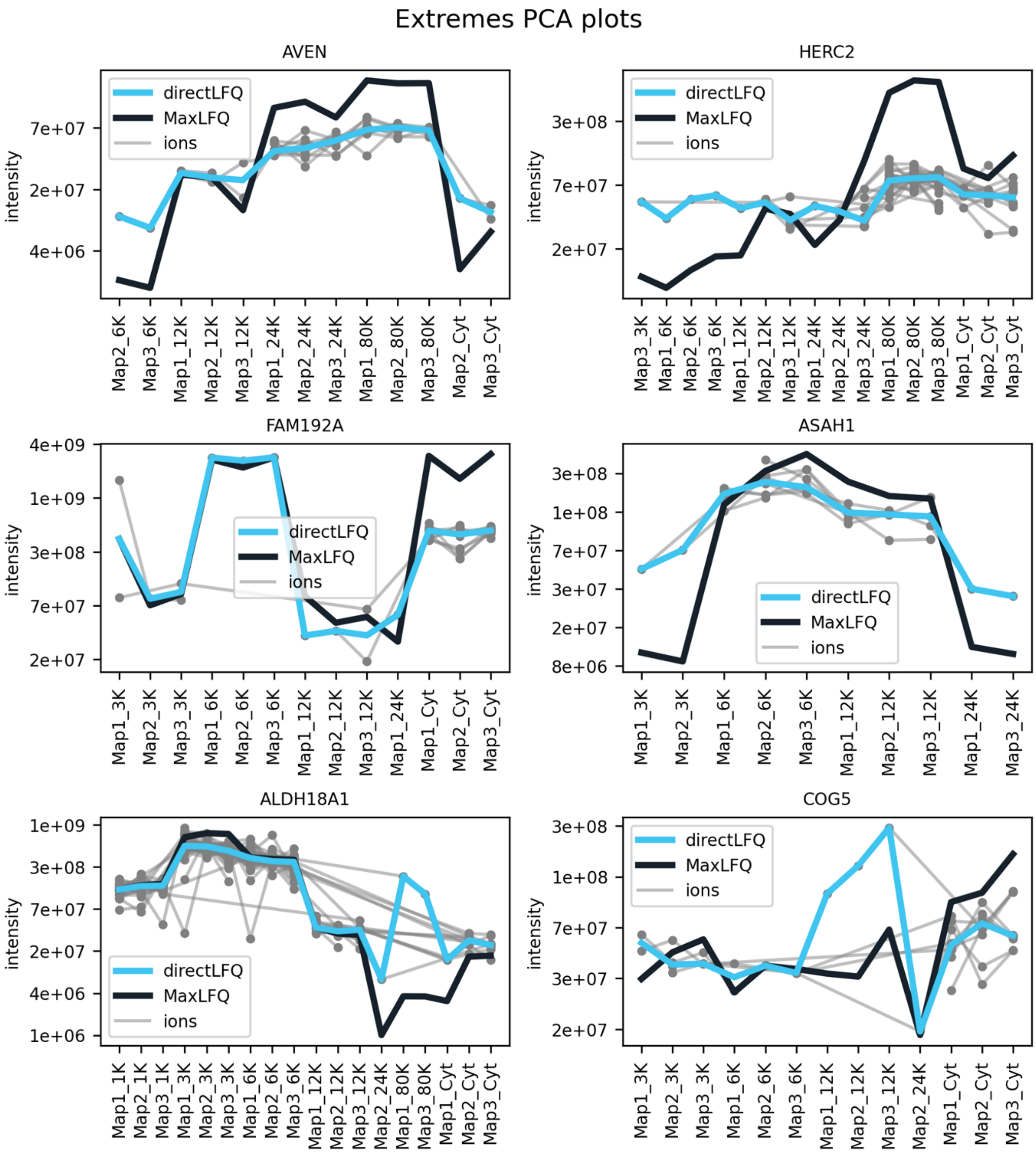

Supplementary Figure 4: Visualizing estimated protein intensity traces for MaxLFQ and directLFQ for different proteins (see main text for a more detailed explanation of the ion intensity plot). The proteins selected were on the edges of the PCA plots displayed in **Figure 4A&B**. We deemed such outlying proteins to be good example cases to see the differences between the directLFQ and the MaxLFQ approach. We see directLFQ (by definition) consistent with the underlying normalized ion data. The MaxLFQ traces are harder to fit towards the normalized ion traces shown here and seem to display stronger variations.
